# Spatial attention-based residual network for human burn identification and classification

**DOI:** 10.1038/s41598-023-39618-0

**Published:** 2023-08-02

**Authors:** D. P. Yadav, Turki Aljrees, Deepak Kumar, Ankit Kumar, Kamred Udham Singh, Teekam Singh

**Affiliations:** 1 Department of Computer Engineering and Applications, GLA University, Mathura, India; 2 Department College of Computer Sci. and Eng., University of Hafr Al-Batin, Hafar Al-Batin, 39524 Saudi Arabia; 3 Department of Computer Science, NIT Meghalaya, Shillong, India; 4 School of Computing, Graphic Era Hill University, Dehradun, 248002 India; 5 Department of Computer Science and Engineering, Graphic Era Deemed to be University, Dehradun, 248002 India

**Keywords:** Biophysics, Cancer, Medical research

## Abstract

Diagnosing burns in humans has become critical, as early identification can save lives. The manual process of burn diagnosis is time-consuming and complex, even for experienced doctors. Machine learning (ML) and deep convolutional neural network (CNN) models have emerged as the standard for medical image diagnosis. The ML-based approach typically requires handcrafted features for training, which may result in suboptimal performance. Conversely, DL-based methods automatically extract features, but designing a robust model is challenging. Additionally, shallow DL methods lack long-range feature dependency, decreasing efficiency in various applications. We implemented several deep CNN models, ResNeXt, VGG16, and AlexNet, for human burn diagnosis. The results obtained from these models were found to be less reliable since shallow deep CNN models need improved attention modules to preserve the feature dependencies. Therefore, in the proposed study, the feature map is divided into several categories, and the channel dependencies between any two channel mappings within a given class are highlighted. A spatial attention map is built by considering the links between features and their locations. Our attention-based model BuRnGANeXt50 kernel and convolutional layers are also optimized for human burn diagnosis. The earlier study classified the burn based on depth of graft and non-graft. We first classified the burn based on the degree. Subsequently, it is classified into graft and non-graft. Furthermore, the proposed model performance is evaluated on Burns_BIP_US_database. The sensitivity of the BuRnGANeXt50 is 97.22% and 99.14%, respectively, for classifying burns based on degree and depth. This model may be used for quick screening of burn patients and can be executed in the cloud or on a local machine. The code of the proposed method can be accessed at https://github.com/dhirujis02/Journal.git for the sake of reproducibility.

## Introduction

Burn is a life-threatening condition that needs early treatment. It is classified into various categories based on its severity and the affected tissues. The most prevalent method for categorization of burns is the "degree" mechanism, which divides burns into three primary categories: first-degree (Superficial dermal), second-degree (Deep dermal), and third-degree (Full thickness) burns. Superficial burn only affects the top layer of the skin (epidermis). The main symptoms include redness, pain, and minor swelling. Healing usually occurs within a few days without scarring^1^. Deep dermal burn affects the epidermis and part of the dermis (the second layer of skin). Symptoms include redness, blistering, severe pain, and swelling. Healing time can vary, and scarring may occur depending on the depth and extent of the burn. Full-thickness burn extends through the entire epidermis and dermis, reaching into the subcutaneous tissue. Symptoms may include a leathery or charred appearance, insensitivity to pain (due to nerve damage), and white or dark brown coloration. Healing is slow and may require skin grafting, and scarring is common. For human burn treatment, first aid can’t be administered to a burn victim before properly diagnosing the injury^2^. The deeper the burn, the more severe the injury. A dermatologist assesses burn severity before grafting is performed. Grafting involves replacing the damaged skin with healthy tissue from an unburned area. After 14–21 days of therapy, a superficial (first-degree) burn will recover. In Table 1, we see how a doctor determines the severity of burns based on the color of the affected areas.

**Table 1:** The classification of human burn skin according to its color.

Burn	Colors	Capillary refill	Scarring	Blisters	Healing
Full thickness	White/Brown/deep red	Absent	Yes	No	Grafting required
Superficial dermal	Red/Pale pink	Brisk 1–2 s	None/Slight color mismatch	Small	Within 14 days
Deep dermal	Blotchy red/white	Sluggish > 2 s/absent	Yes	±	Grafting required

The manual burn diagnosis process requires expert involvement, making the process time-consuming and expensive. Dermatological experts employ fluorescence fluorometry, fluorescence, and ultrasound imaging to predict burn depth, achieving diagnostic accuracy between 50 and 80%^3^. Deep-dermal burns affect the second skin layer, while full-thickness burns penetrate the third layer and often involve damaged tissues, muscles, and scarring, significantly impacting a patient’s life. Effective treatment for burn scars is essential, and doctors utilize anti-scarring techniques^4^. The severity of burns can also have long-term negative consequences for patients^5^. Past studies have employed machine learning methods for burn diagnosis, which typically involve preprocessing burnt images to downsize and reduce noise. Handcrafted texture and shape features are manually extracted for training and classifying burn types, but this approach requires a small dataset and specialized expertise, leading to potential errors that reduce model performance.

In contrast, deep learning models can automatically learn features through their layers, demonstrating promising capabilities for medical image recognition in recent years^6^. However, the deep CNN model performance depends on the dataset size and model architecture^7^. Previous research utilizing deep CNN techniques has shown improved performance^8–13^. Yet, some deep learning models with few layers and limited training datasets have led to suboptimal performance for burn diagnosis. Models like ResNeXt, AlexNet, and VGG16 were computationally expensive and did not achieve remarkable accuracy in burn diagnosis. For burn degree categorization, ResNeXt, AlexNet, and VGG16 achieved classification accuracies of 84.31%, 70.57%, and 76.32%, respectively, similar to the manual approach. We propose a spatial attention-based model called BuRnGANeXt50 to address these challenges. This model utilizes a feature map divided into categories, highlights channel dependencies within each class, and builds a spatial attention map to improve classification accuracy. Efficiently capturing information regarding the depth of the burn region is crucial for severity assessment and surgical recommendations for grafting. The proposed model demonstrates excellent performance in quickly screening different types of burns while also being computationally efficient.

The significant contribution of the manuscript is as follows.

1. The proposed BuRnGANeXt50 is a residual network that takes less computation time than ResNeXt. Since ReNext has 23 × 10^6^ and our model has 5 × 10^6^ neurons
2. Two-channel maps are separated into categories, and the channel dependencies between them are highlighted. Meanwhile, a spatial attention map is built from the spatial relationships between features
3. The training and validation loss on BIP_US Database is significantly less, which confirms that the proposed model is sensitive for burn diagnosis

The rest of the paper is organized as follows:

“Literature review” Section describes a study involving a detailed review of human burns. At the same time, “Proposed method” section describes the architecture of the BuRnGANeXt50 model. “Results” section describes the experimental procedures for the diagnosis of urn based on degree and depth. Finally, in “Discussion” section, the comparative study of different models and BuRnGANXt50 is described in detail.

## Literature review

To segment renal tissue and identify immunological (CD3 +) inflammatory cells, Hermsen et al.^14^ developed two CNNs models. Human evaluation of the Banff lesion types was compared with automated measurement of glomeruli, interstitial fibrosis, and (total) inflammation, and strong correlations were found. Long-term changes in estimated glomerular filtration rate (eGFR) are inversely related to inflammation inside scarred regions, according to automated and visual examination of a small cohort^14^. The machine learning technique used by Abubakar et al.^15–17^ classifies the human burn using the African dataset. Burn healing times may be used to predict burn depth. Specifically, they used One-versus-One SVM to examine the efficacy of leveraging deep features obtained from a pretrained model to address a multiclass problem. Relevant discriminating characteristics of the images were obtained using VGG16 and pretrained ResNet50. With VGG16 features (VggFeat16), the proposed method achieved a prediction accuracy of 85.67%, whereas The ResNet50 model achieved a maximum classification accuracy of 95.43%^15^.

Furthermore, Suha et al.^18^ proposed a Deep Convolutional Neural Network (DCNN)-based model with transfer learning and fine-tune for assessing skin burn degree from real-time burn photos of patients. The design utilizes several convolutional layers and hyperparameter tuning for feature extraction and image classification into three distinct classes. The traditional approach, which used digital image processing and regular machine learning classifiers, was also tested and evaluated for this multiclass classification problem^18^. Abubakar et al.^15–17^ observed that 90.45% of the time, it was possible to identify the different types of burns correctly. This study lays the groundwork for future investigations, notably in healthcare, to focus on how racial feature representations may be integrated with training data to produce effective and widely used diagnostic tools^16^.

The convolutional neural network-based approach for body part burn image identification Chauhan et al. (2020) has introduced a deep CNN model that can be used to develop more effective computer-assisted burn diagnostic tools by combining non-burn images with the body part-specific burn severity rating model. The burn image body part classification (BI-BPC) and Body Part-specific Burn Severity Assessment Model utilized deep convolutional neural networks for evaluating the two labelled burn image datasets (BPBSAM). Using BI-BPC and ResNet50 for feature extraction in severity evaluation shows maximum efficiency^19^. Pabitha et al.^20^ presented a hybrid model combining DenseMask RCNNs with transfer learning to classify skin burns accurately. They engage in dense pose estimation^20^ to split the burn zone, classify it into varying degrees, and compute the burned depth according to the severity of the lesion.

Khan et al.^21^ collected burn images from people of diverse ages and ethnicities. It was nearly impossible to collect images from healthcare facilities due to ethical concerns. Using image mining and DCNN classification, a method is described for segmenting damaged skin and calculating burn depth. A hybrid segmentation method eliminates the background of a picture of burned flesh. The burn depths were classified using a DCNN with an accuracy of 79.4%^21^. Wu et al.^22^ developed a convolution neural network (CNN) method for burn image recognition. The experimental results in this work show that the CNN based model can effectively classify and detect burn areas^22^.

Recent research by Khan et al.^21^ discriminated between mild and severe burn symptoms using preprocessing and image-down sampling methods. Otsu’s technique is used to remove the scorched area from the image. Their method categorizes burns as first, second, or third-degree. The performance of the model can be better using more complex CNN models and bigger data sets^21^. Recently, Rostami et al.^23^ developed a deep learning model for diagnosing human burns. Their method employs a deep CNN model for feature extraction, whereas SVM is employed for classification. The fivefold cross-validation method achieves 87.7% accuracy in multiclass classification, whereas the binary class achieves 94.28% accuracy^23^. Some of the recent method used for burn diagnosis using ML and DL has been shown in Table 2.

**Table 2:** Summary of the recent work using ML and DL.

Authors	Method	Dataset Size	Accuracy
Chang et al.^48^	ResNet101	4991 images	98.84%
Boissin et al.^24^	CNNs	1105 images	87.2%
Anisuzzaman et al.^25^	YOLO V3	1010 images	93.9%
Khani et al.^26^	SVM	40 images	94.7%
Anisuzzaman et al.^27^	WIC	1088 images	97.12%
Lee et al.^28^	BurnNet	586 images	99%
Kumar et al.^29^	DeepLabV3 + SE	1841 images	96.3%
Santos et al.^30^	V19V16ResDenIn	8250 images	95.04%
Eldem et al.^31^	AlexNet	2090 images	95.48%
Ahsan et al.^32^	VGG16	1459 images	98.48%

## Proposed method

Accurate diagnosis of human burns requires a sensitive model. ML and DL are commonly employed in medical imaging for disease diagnosis. ResNeXt, AlexNet, and VGG16 are state-of-the-art deep-learning models frequently utilized for medical image diagnosis. In this study, we evaluated and compared the performance of these models for diagnosing burn images. However, these models showed limited effectiveness in accurate diagnosis of burn degree and distinguishing grafts from non-grafts.

ResNeXt, a deep residual model, consists of 50 layers, while AlexNet and VGG16 are sequential models with eight and 16 layers, respectively. These layers extract features from the burned images during the model’s training process. Unfortunately, distinguishing between deep dermal and full-thickness burns can be challenging, as they share similar white, dark red, and brown colors. Consequently, highly delicate and stringent methods are required for accurate differentiation. AlexNet and VGG16, being sequential models, mainly extract low-level features, whereas ResNeXt excels in extracting high-dimensional features. A limitation is that these models can only learn positive weight features due to the ReLu activation function. This constraint may hinder their ability to precisely identify critical burn characteristics. The DL models, AlexNet, ResNeXt, VGG16, and InceptionV3 are widely used for medical image diagnosis, however, these models encounter challenges in accurately categorizing burn degrees and differentiating grafts from non-grafts. Finding effective ways to handle these challenges and improve feature extraction could lead to more sensitive and reliable burn diagnosis models.

**Figure 1:**
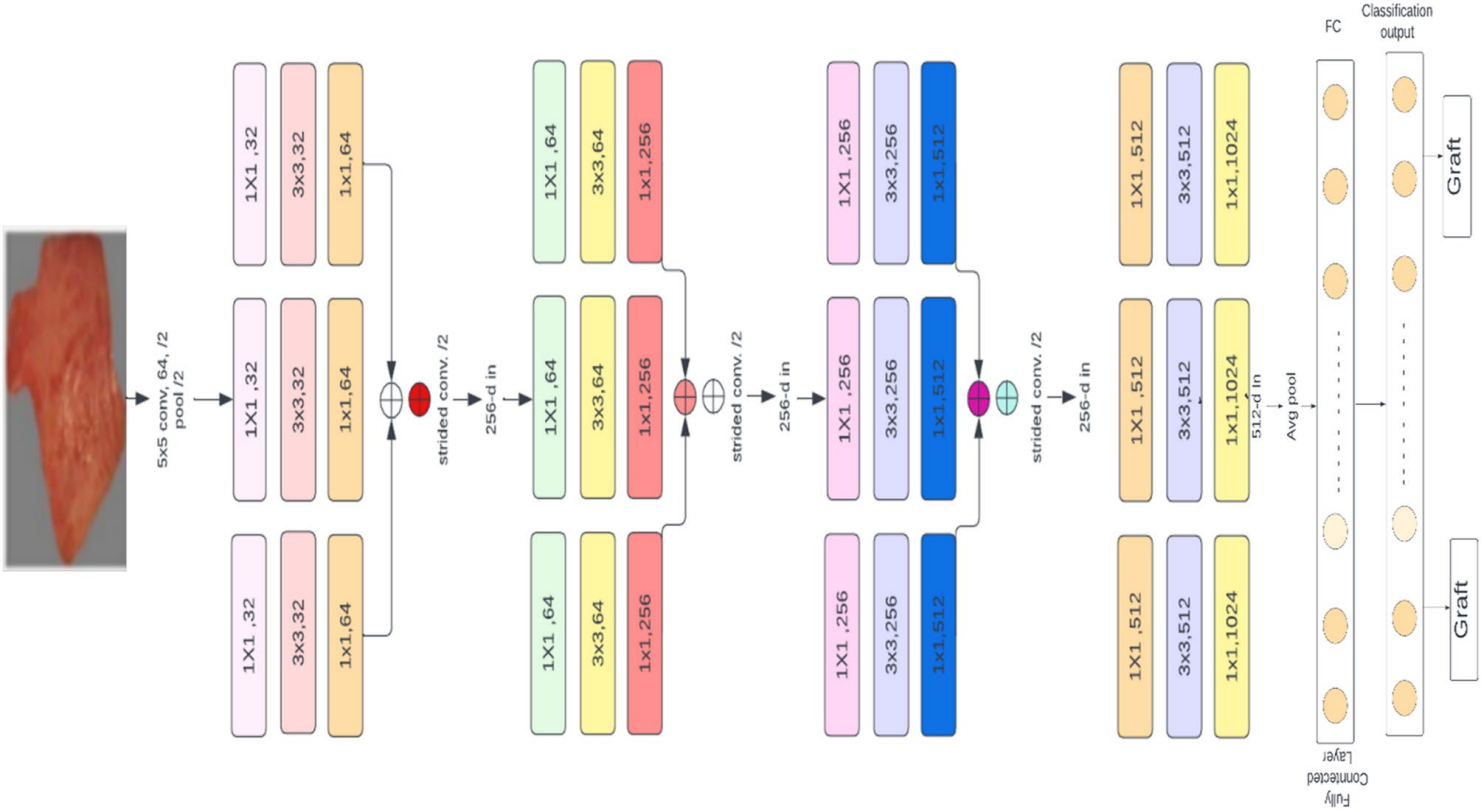
Topology of BuRnGANeXt50 for human burn diagnosis.

The ResNeXt model^33^ influenced the BuRnGANeXt50 model. To construct a BuRnGANeXt50 model, the original ResNeXt model’s topology is modified. Moreover, the original ResNeXt was created to classify images into several categories with high computation costs. In this study, the method performs a multiclass and binary class classification task. Multiclass classification is used to assess burn severity based on burn depth. After that, based on depth, burns may be broken down into two distinct types: graft and non-graft. Reducing the first layer filter size from 7 × 7 to 5 × 5 is the first change to the original ResNext model’s design because a larger filter size resulted in lower pixel intensity in the burnt region. This has led to a rise in the frequency of spurious negative results for both grafts and non-grafts. Furthermore, the convolution sizes of Conv1, Conv2, Conv3, Conv4, and Conv5 are also changed to reduce the computation cost while maintaining cardinality. Furthermore, we applied Leaky ReLu instead of the ReLU activation for faster model convergence. Table 2 also shows that conv2, conv3, and conv4 are shrinking in size. After implementing all modifications, neurons decreased from 23 × 10^6^ to 5 × 10^6^, as shown in Table 3. The detailed architecture of the proposed model is shown in Fig. 1.

**Table 3:** BuRnGANeXt50 model versus original topology.

Stage	ResNeXt-50			BuRnGANeXt50		
Conv1	7 × 7, 64, number of strides 2			5 × 5, 256, Number of strides 2		
Conv2	3 × 3 max pool, stride 2			3 × 3 max pool, stride 2		
	1 × 1, 128	C = 32	X3	1 × 1, 32	C = 32	X3
	3 × 3, 128			3 × 3, 32		
	1 × 1, 256			1 × 1, 128		
Conv3	1 × 1, 256	C = 32	X4	1 × 1, 64	C = 32	X4
	3 × 3, 256			3 × 3, 64		
	1 × 1, 512			1 × 1, 256		
Conv4	1 × 1, 512	C = 32	X6	1 × 1, 256	C = 32	X6
	3 × 3, 512			3 × 3, 256		
	1 × 1, 1024			1 × 1, 512		
Conv5	1 × 1, 1024	C = 32	X3	1 × 1, 512	C = 32	X3
	3 × 3, 1024			3 × 3, 512		
	1 × 1, 2048			1 × 1, 1024		
#params	23 × 10^6^			5 × 10^6^

**Figure 2:**
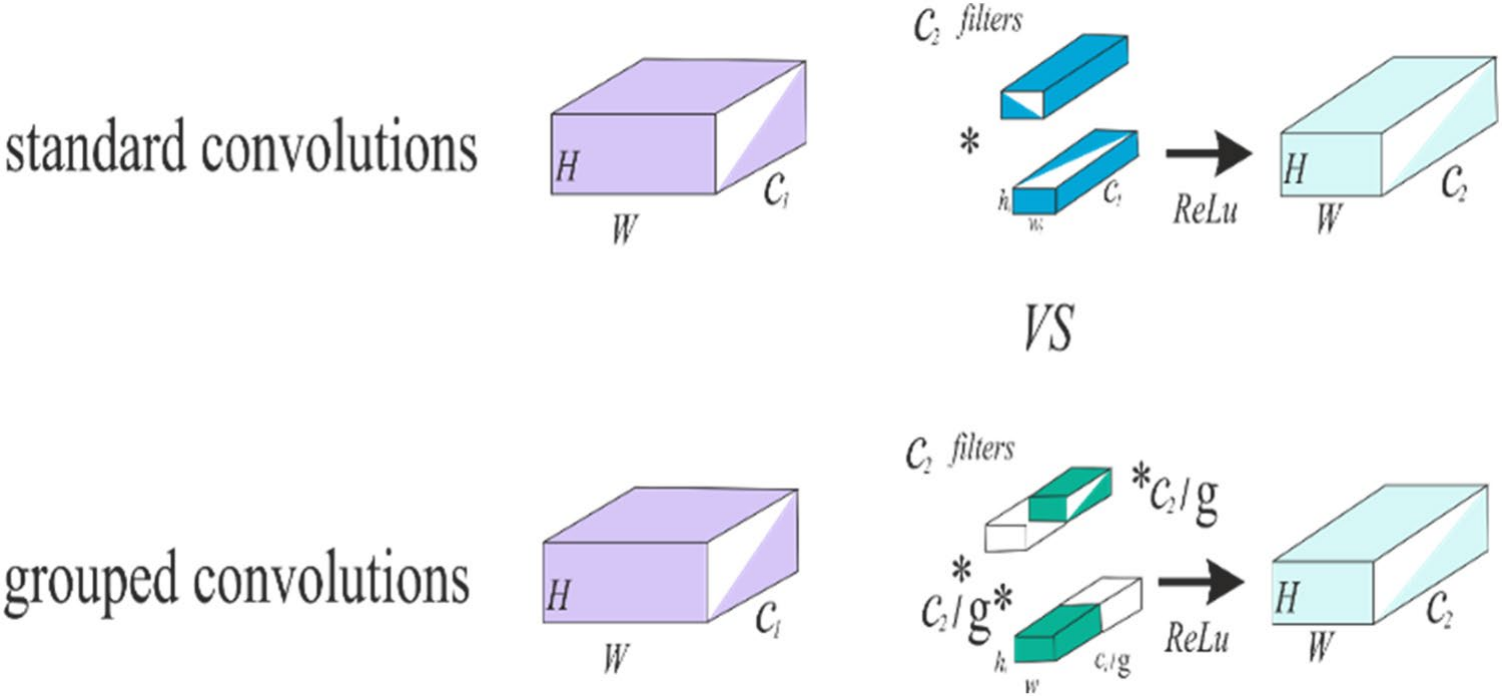
The pooling layers are convolutions in a grouped manner.

This model has several essential building blocks, including convolution, residual, ReLU, activation, softmax, and flattened layer. The results of groups’ convolution of neurons inside the same kernel map are summed together by pooling layers, which reduce the input dimensionality and enhance the model performance. The pooling units in the proposed model constitute a grid, with each pixel representing a single voting location, and the value is selected to gain overlap while reducing overfitting. Figure 2 describes the structure of the model’s convolution layer. Polling units form a grid, each pixel representing a single voting place being centered $z \times z$. In the provided model, we employ the standard CNN with parameters set to $S = z$, but we add a charge of $S < z$ to increase overlap and decrease overfitting^34^. The proposed architecture was developed to handle the unique issues of burn diagnosis, emphasizing decreasing overfitting and enhancing model accuracy.

The inner dot product is an essential part that neurons perform for the foundation of an artificial neural network’s convolutional and fully connected layers. The inner dot product may compute the aggregate transform, as illustrated in Eq. (1).

1 $$\mathop \sum \limits_{i = 1}^{K} w_{i} \rho_{i}$$

represents the neuron’s k-channel input vector. Filter weight is given by $w_{i}$for i-the neurons. This model replaces the elementary transformations with a more generic function $\left( {w_{i} \rho_{i} } \right)$. By expanding along a new dimension, this generic function reduces depth. This model calculates the aggregated transformations as follows:

2 $${\Im }\left( \rho \right) = \mathop \sum \limits_{i = 1}^{{\mathbb{C}}} \Upsilon_{i} \left( \rho \right)$$

**Figures 3:**
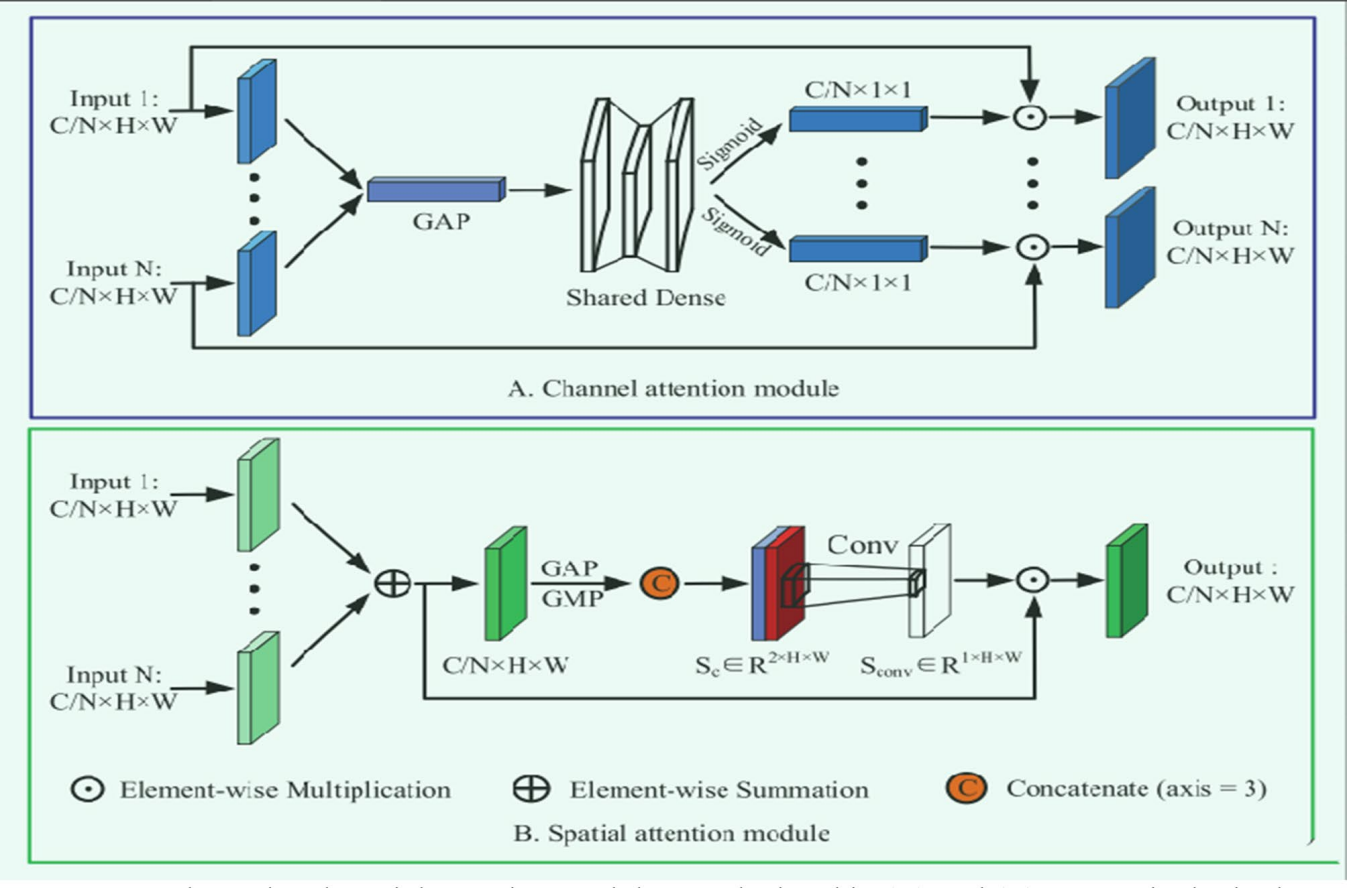
Channel and spatial attention modules are depicted in (**A**) and (**B**), respectively, in these schematic illustrations.

The function $\Upsilon_{i} (\rho )$ is arbitrarily defined. $\Upsilon_{i}$ project $\rho$ into low-dimensional embedding and then change it, similar to a primary neuron. ${\mathbb{C}}$ represents the number of transforms to be summed in Eq. (2). ${\mathbb{C}}$ is known as cardinality^35^. As the residual function, Eq. (2)‘s aggregated transformation serves^36^. (Fig. 3):

3 $$x = \rho + \mathop \sum \limits_{i = 1}^{{\mathbb{C}}} \Upsilon_{i} \left( \rho \right)$$

where $x$ is the model’s predicted result.

Finally, at the top of the model a flattened and a global average pooling is added. The Softmax activation classifies burn into binary and multiclass. The softmax optimizer uses the exponent of each output layer to convert logits to probabilities^37^. The vector $\Phi$ is the system input, representing the feature set. Our study uses k classification when there are three levels of burn severity (k = 3) and two levels of graft versus non-graft (k = 2). For predicting classification results, the bias $W_{0} X_{0}$ is added to each iteration.

4 $$p(\rho = i|\Phi^{\left( j \right)} ) = \frac{{e^{{\Phi^{\left( j \right)} }} }}{{\mathop \sum \nolimits_{i = 0}^{k} e^{{\Phi_{k}^{\left( j \right)} }} }}$$

5 $${\text{In}}\;{\text{which}}\;\Phi = W_{0} X_{0} + W_{1} X_{1} + \ldots + W_{k} X_{k}$$

### Residual attention module

The residual attention block, which allows attention to be routed across groups of separate feature maps, is shown in Fig. 3. Furthermore, the channel’s extra feature map groups combine the spatial information of all groups via the spatial attention module, boosting CNN’s capacity to represent features. It comprises feature map groups, feature transformation channels, spatial attention algorithms, etc. Convolution procedures can be performed on feature groups, and cardinality specifies the number of feature map groups. A new parameter, "S," indicates the total number of groups in the channel set^38^ and the number of subgroups in each of the N input feature groups. A channel scheduler is a tool that optimizes the processing of incoming data through channels. This method transforms feature subsets. G = N * S is the formula for the total number of feature groups.

Using Eq. (6), we conduct an essential feature modification on subgroups inside each group after channel shuffling.

6 $$g\left( {r,i,j} \right) = \left[ {\begin{array}{*{20}c} {\cos \frac{r\pi }{2}} & { - \sin \frac{r\pi }{2}} \\ {\sin \frac{r\pi }{2}} & {\cos \frac{r\pi }{2}} \\ \end{array} } \right]\left[ {\begin{array}{*{20}c} i \\ j \\ \end{array} } \right]$$

Here $0\le r<4,\left(i,j\right)$ stands for the original matrix’s coordinates. K represents the 3 × 3 convolution of the bottleneck block, and Output is written as $y_{s}$. Then, for each $x_{s}$ input

we have:

7 $$y_{s} = \left\{ {\begin{array}{*{20}c} {K\left( {g_{r} \left( {x_{s} } \right)} \right)r,} & {s = 0} \\ {K\left( {g_{r} \left( {x_{s} } \right)} \right) \odot y_{0} } & {0 < r = s < 4} \\ \end{array} } \right.$$

$g\& r$ here represents the input $x_{s}$. “$\odot$” corresponds to element multiplication in the matrix’s related feature transformation. Features of x being transformed are shared across the three 3 × 3 convolution operators K.

### Channel and spatial attention modules

Semantic-specific feature representations can be improved by exploiting the interdependencies among channel graphs. We use the feature map’s channels as individual detectors. Figure 3A depicts how we send the feature map of the $no\in \mathrm{1,2},...,N$ group ${G}^{no}\in {R}^{C/N\times H\times W}$ to the channel attention module. As a first step, we use geographic average pooling (GAP) to gather global context information linked to channel statistics^39^. The 1D channel attention maps ${C}^{no}\in {R}^{C/N}$ are then inferred using the shared fully connected layers.

8 $$C^{n} = D_{sigmoid} \left( {D_{{{\text{Re}} LU}} \left( {GAP\left( {G_{n} } \right)} \right)} \right)$$

$"{D}_{sigmoid}and{D}_{\mathit{Re}LU}"$ represents a fully linked layer that uses both "Sigmoid" and "ReLU" as activation functions. At last, Hadamard products are used to infer a group’s attention map and the corresponding input features. Then the components from each group are weighted and added together to produce an output feature vector. The final channel attention map

9 $$C \in R^{C/N \times H \times W} C = \mathop \sum \limits_{n = 1}^{N} \left( {C^{n} \odot G^{n} } \right)$$

Each group’s 1 × 1 convolution kernel weight is multiplied by the 3 × 3 kernel weight from the subgroup’s convolutional layer. The global feature dependency is preserved by adding the group’s channel attention weights, which all add up to the same value.

A spatial attention module is used to synthesize spatial links and increase the spatial size of associated features. The channel attention module is separate from that component. The spatial information of feature maps is first aggregated using global average pooling (GAP) and maximum global pooling (GMP)^39^ to obtain two distinct contextual descriptors. Next, by joining $GAP(C)\in {R}^{1\times H\times W}andGMP(C)\in {R}^{1\times H\times W}$ connect to get ${S}_{c}\in {R}^{2\times H\times W}$.

10 $$S_{c} = GAP\left( C \right) + GMP\left( C \right)$$

The plus sign “+”denotes a linked feature map. The regular convolutional layer retrieves the spatial dimensional weight information to round things out. $S_{conv}$ Final spatial attention map $S\in {R}^{C/N\times H\times W}$ is obtained by element-wise multiplying the input feature map $C$ with itself.

11 $$S = Conv_{3 \times 3} \left( {S_{C} } \right) \odot C$$

$"Con{v}_{3\times 3}"$ means regular convolution, while "Sigmoid" denotes the activation function.

### Local response normalization

Leaky ReLU activation-based deep learning models do not rely on input normalization for saturation. Neurons in this model are more efficient at learning from negative inputs. Despite this, neural activity is calculated ${\alpha }_{u,v}^{i}$ At a point $(u,v)$ by using the kernel $i$, which facilitates generalization. The ReLU nonlinearity is then implemented. The ReLU nonlinearity is then implemented. The response normalized ${\alpha }_{u,v}^{i}$ is determined using the provided Eq. (12).

12 $$b_{u,v}^{i} = \frac{{\alpha_{u,v}^{i} }}{{\left( {t + \alpha \mathop \sum \nolimits_{j - \max (0,i,n/2)}^{\min (N,1,i + n/2)} (\alpha_{u,v}^{j} )^{2} } \right)^{\beta } }}$$

where $N$ are the total number of layers and $t,\alpha ,n,\beta$ are constants? This $\sum {}$ is computed for each of the $n$ neighboring^40^. We trained the network using a $100 \times 100 \times 3$ picture and the original ResNeXt CNN topology’s cardinality hyper-parameter ${\mathbb{C}}=32$. The algorithm of the proposed method is shown below.

Algorithm of the proposed method.
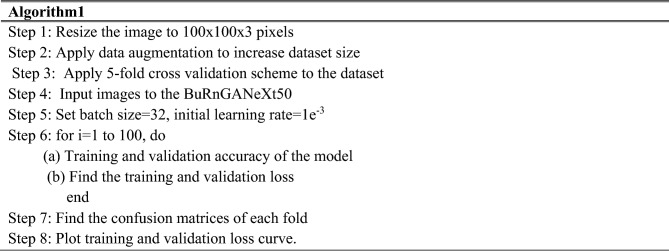


### Ethical approval

All authors contributed to the conception and design of the study. All authors read and approved the final manuscript.

## Results

### Dataset

**Figure 4:**
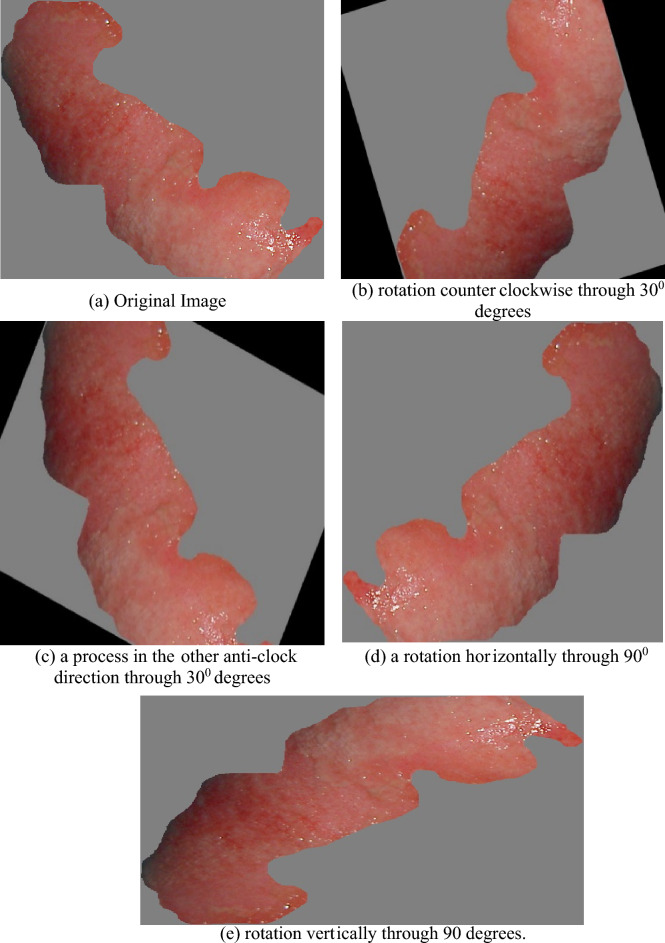
(**a**) original, (**b**) rotation counterclockwise through 30^0^ degrees, (**c**) a process in the other anti-clock direction through 30^0^ degrees, (**d**) a rotation horizontally through 90 degrees, and (**e**) rotation vertically through 90 degrees.

The dataset includes images of human burns having several depths (super-dermal, deep-dermal, and full-thickness) and types (graft, non-graft). The University of Seville’s (Spain) Signal Theory and communications department’s biomedical image processing (BIP) Group at the Virgen del Roco Hospital (Spain) collected images. There are 94 images with varying sizes in the original dataset compiled from^41^. Four types of data augmentation techniques were applied to the burn image: horizontal flip, vertical flip, rotation through 30°, and rotation through 30°. Finally, 6000 images retained the augmented dataset. Figure 4a–e show the augmented images of the modified dataset.

The proposed method is implemented on Nvidia GeForce GTX TITAN X GPU using Python 3.8 and Tensor Flow 2.0. The BuRnGANeXt50 is trained with images of batch size 32 and an initial learning rate of 1e^-3^ on the Windows 10 operating system.

### The mathematical technique of model performance analysis

The system’s efficacy is evaluated using a confusion matrix and its f1-score, accuracy, precision, recall, sensitivity, and specificity values. They are calculated using the true positive (TP), false positive (FP), false negative (FN), and true negative (TN) indicators (True Negative).

13 $$Acc = \frac{TP + TN}{{TP + TN + FP + FN}}$$

14 $$PRE = \frac{TP}{{TP + FP}}$$

15 $$RE = \frac{TP}{{TP + FN}}$$

16 $$f1 - score = 2{*}\frac{{PRE{*}RE}}{PRE + RE}$$

17 $$Sen = \frac{TP}{{TP + FN}}$$

18 $$Spec = \frac{TN}{{TP + TN}}$$

where $TN=$ The model labels it as unfavorable since it is a negative number. $TP=$ It is a genuine positive value, and the model also classifies it as positive. $FP=$ The model incorrectly interprets a negative value as positive. $FN=$ It is a positive number and a negative category of models.

### Classification of burn degrees (multiclass)

**Figure 5:**
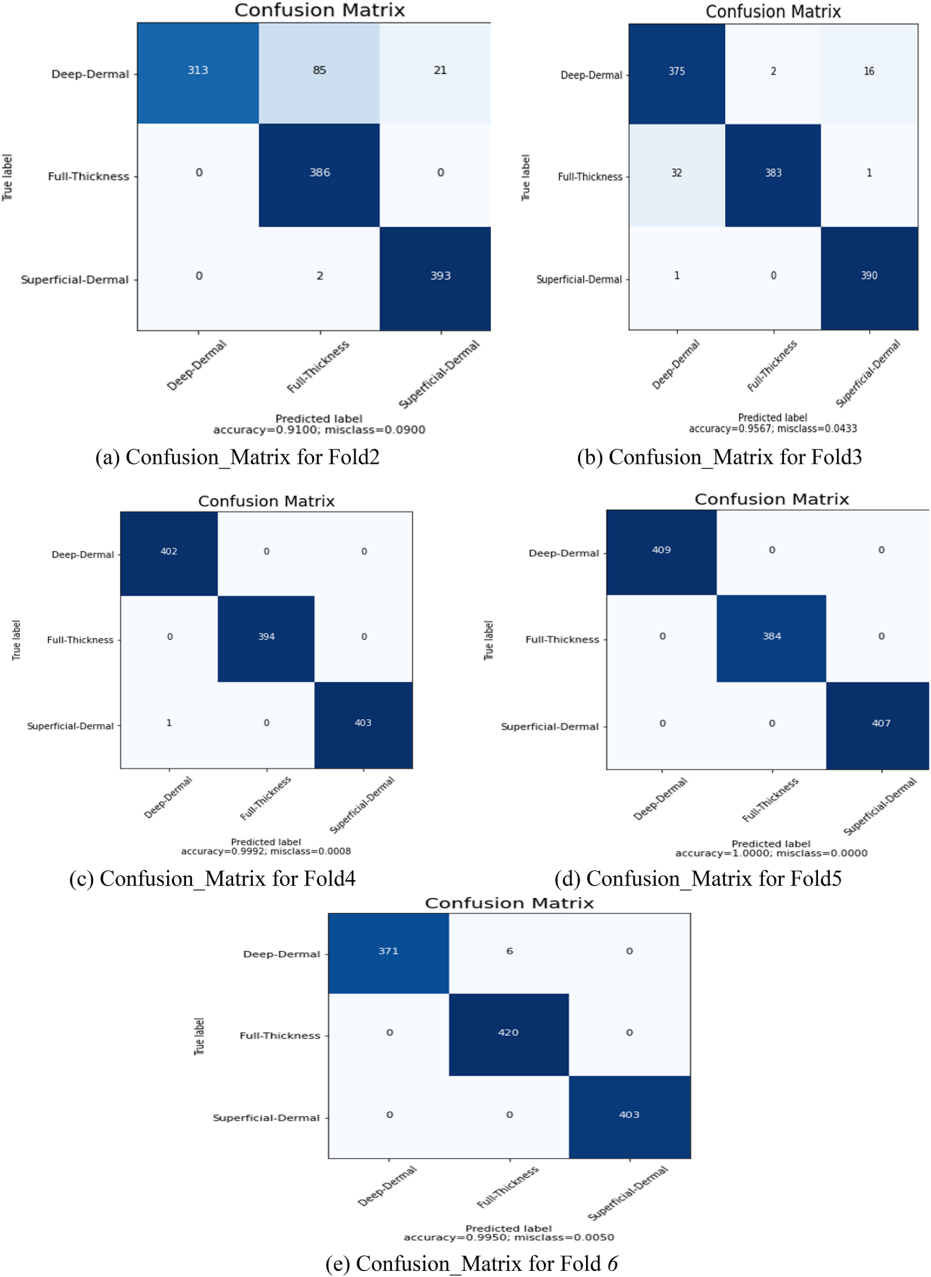
(**a**) Confusion_Matrix for Fold1, (**b**), Confusion_Matrix for Fold2, (**c**), Confusion_Matrix for Fold3, (**d**), Confusion_Matrix for Fold4 (**e**), and Confusion_Matrix for Fold5.

The extended dataset^41^ comprises images of superficial burns, deep burn, and full-thickness burns. To ensure an unbiased model, a fivefold cross-validation is employed. In this process, the dataset is divided into 80% for training and 20% for validation, with each fold using different partitions. The training is conducted with an initial learning rate of 1e-3, and the input image size is downsampled to 100 × 100 pixels. The model undergoes 100 training iterations, utilizing a mini-batch size of 32. After training on each of the five test data folds, confusion matrices (CM) are generated, as depicted in Fig. 5. The obtained confusion matrices are shown in Fig. 5a–e. The results of the provided BuRnGANeXt50 model for each fold are shown in Table 4. Average values for the model’s sensitivity, specificity, F1-score, recall, and accuracy were 97.25%, 97.22%, 97.2%, 98.65%, and 97.17%. Classification accuracy for burns of varying depths in the skin (superficial, deep, and full thickness) is more than 98% using this approach.

**Table 4:** The multiclass classification effectiveness measures of the BuRnGANeXt50 model.

Folds	Performance metrics (%)					
	Precision	Recall	F1-score	Sensitivity	Specificity	Accuracy
Fold1	91.00	91.01	91.00	91.00	95.40	94.00
Fold2	95.66	95.66	95.66	95.66	97.83	97.11
Fold3	99.92	99.92	99.92	99.92	99.95	99.94
Fold4	100	100	100	100	100	100
Fold5	99.5	99.5	99.5	99.5	99.75	99.66
Average	97.22	97.22	97.22	97.22	98.61	98.14

**Figure 6:**
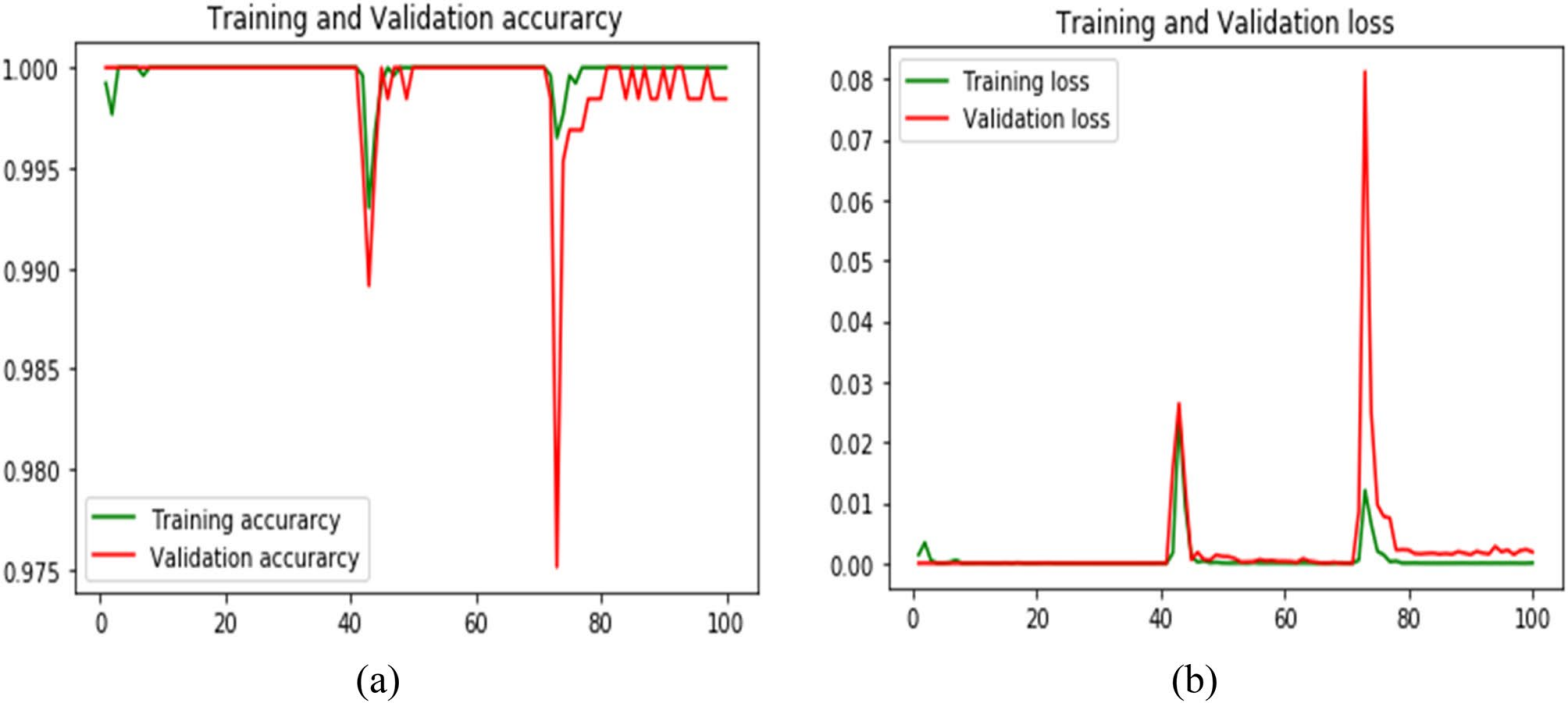
Proposed multiclass classification training and validation accuracy and loss.

The model training and validation loss are displayed in Fig. 6. Figure 6a shows that the model has a training accuracy of around 100% and a validation accuracy of over 98%. Figure 6b shows that after 80 iterations, the training loss and validation loss have reduced to almost zero.

### Burn’s graft and non-graft diagnosis (binary classification)

**Figure 7:**
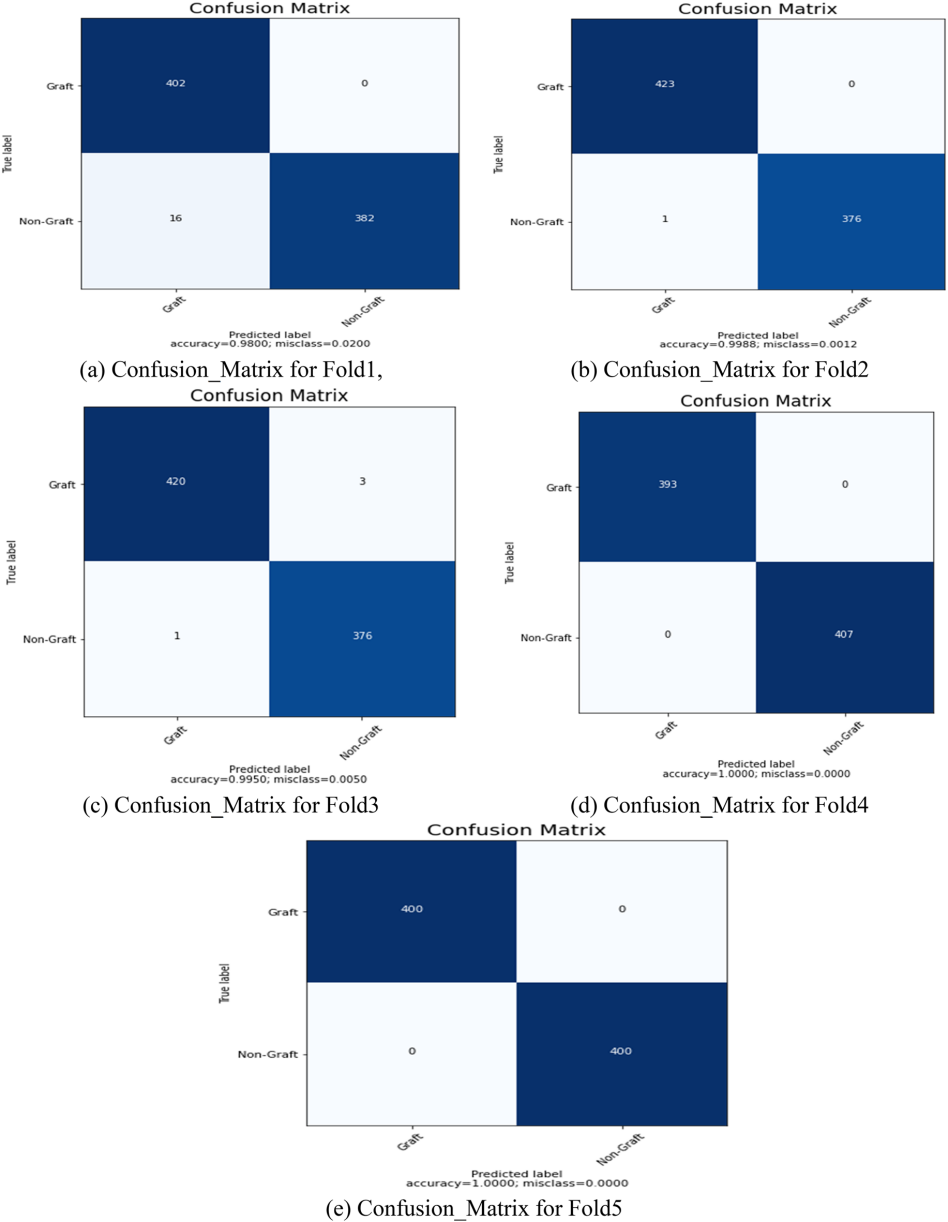
(**a**) Confusion_Matrix for Fold1, (**b**) Confusion_Matrix for Fold2, (**c**) Confusion_Matrix for Fold3, (**d**) Confusion_Matrix for Fold4, and (**e**) Confusion_Matrix for Fold5.

For further diagnosis, the degree of burn is required. The severity of a burn can be determined by depth^42^. A doctor uses the grafting procedure to replace the burned skin on a patient’s body. Often, grafting is necessary for full-thickness and severe burns^43^. The improved burn dataset consists of graft and non-graft. The grafts represent full-thickness and deep dermal burns, whereas non-grafts represent superficial burns. Four thousand images of human burns were utilized for the binary classification. Moreover, fivefold cross-validation was performed on the dataset. The data set is split into two halves, 80% and 20%, using a fivefold cross-validation. The training set uses 80% of the data for every fold, while the validation set uses 20%. The input image is scaled to 100 × 100 × 3 pixels, and the model’s initial learning rate is set to 1e-3. After that, a mini-batch size of 32 and 100 iterations was used to train the model. Figure 7 displays the confusion matrices (CM) acquired after training for each of the 5 test data folds. Figures 7a–e are five confusion matrices obtained for graft and non-graft. The performance measures of the BuRnGANeXt50 model across all folds are shown in Table 4. The model also has a sensitivity of 99.14%, a specificity of 99.84%, and an accuracy of 99.48% when classifying data into binary categories.

**Figure 8:**
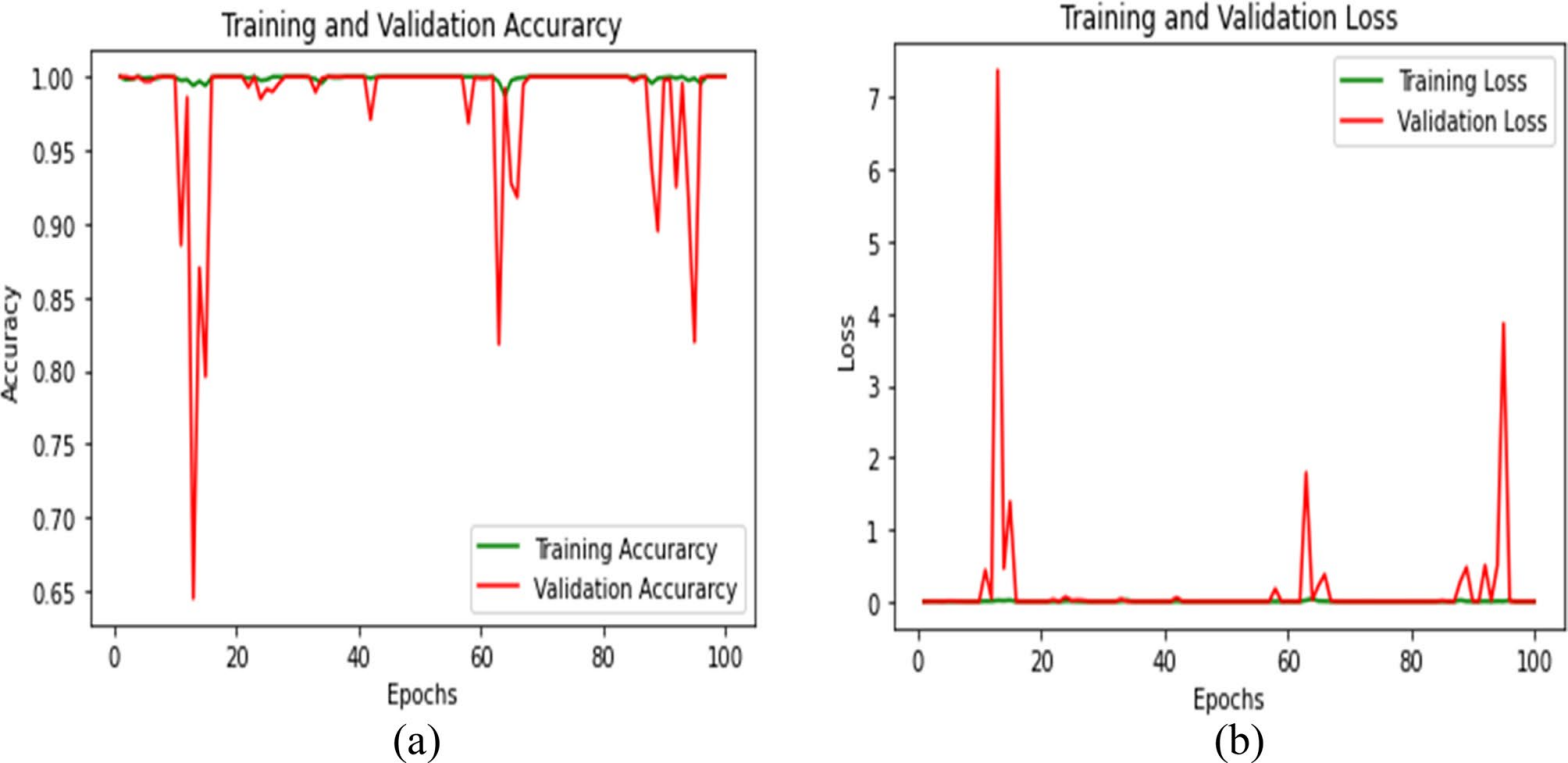
Computation analysis of training and validation of accuracy (**a**) and tanning and validation loss (**b**).

Classification accuracy of the training and validation as well as loss, are displayed in Fig. 8. After 45 iterations, the accuracy during training is close to 100% (Fig. 8a). Similarly, the model’s training and validation losses are near 0 and saturated after 45 iterations (Fig. 8b). Table 5 depicts the performance of all 5 folds of the proposed BuRnGANeXt50 model.

**Table 5:** performance metrics for binary class classification of the BuRnGANeXt50 model.

Folds	Accuracy	Recall	Precision	Sensitivity	Specificity	F1-score
Fold1	98.00	96.17	100	96.17	100	98.05
Fold2	99.88	99.76	100	99.76	100	99.88
Fold3	99.50	99.76	99.29	99.76	99.21	99.53
Fold4	100	100	100	100	100	100
Fold5	100	100	100	100	100	100
Average	99.48	99.14	99.86	99.14	99.84	99.49

## Discussion

Automated methods for human burn diagnosis, utilizing deep learning, machine learning, and transfer learning, have been explored in various studies. For instance, Abubakar et al.^15–17^ employed deep transfer learning with ResNet50 and VGG16 to extract visual patterns from a dataset of 2080 RGB images containing healthy skin and burns. Their proposed technique achieved a maximum prediction accuracy of 95.43% using ResNet50 and 85.63% with VggFeat16. Similarly^44^, utilized ResNet101 for burn skin prediction, achieving a 95.9% accuracy rate.

Another study by Yadav et al.^40^ focused on diagnosing burns and categorizing them as graft or non-grafts, achieving an accuracy of 82%. Abubakar et al.^15–17^ employed transfer learning to classify skin as burnt or healthy, reaching 99.3% accuracy with the Caucasian dataset and 97% with the African dataset.

Machine learning models for evaluating burn severity were presented by Shin et al., achieving a 70.0% accuracy rate on an unlabelled dataset of 170 images learned via self-supervised learning techniques. Rahman et al.^46^ suggested a vision-based method for identifying burns on the skin, with their SVM model achieving a maximum accuracy of 93.33%.

Despite their usefulness, these approaches have drawbacks, such as high computation costs and decreased efficiency in predicting grafts and non-grafts. To address these challenges, we proposed BuRnGANeXt50, an attention-based residual network that is less expensive and more efficient than ResNeXt. The suggested approach optimizes the convolution size and kernel size, and Leaky ReLu activation is employed to accelerate convergence. A channel and spatial attention module are also included to improve the local feature co-relationship. The existing methodologies for diagnosing burns using diverse datasets are summarised in Table 6. In Table 6, we can notice that the automatically trained classification models outperform compared to manually extracted feature classification models.

**Table 6:** Past work on the diagnosis of human burns using various ML and DL.

Study	Model	Classification	Dataset Size	Accuracy (%)
Abubakar et al.^15^	Pre-trained ResNet50	Binary Class	2080 images	95.43
	Pre-trained VGG16			85.63
Smith et al.^16^	Pre-trained ResNet50	Binary Class	1360 images (Caucasians dataset)	99.3
			540 images (African dataset)	97.1
Ugail et al.^44^	ResNet101	Binary Class	1360 images	95.9
Buhar et al.^17^	VGG-Face	Binary Class	1420 images	95.208
Yadav et al.^40^	SVM	Binary Class	94 segmented images	82.43
Shin et al.^45^	SSL	Multiclass	170 images	70.0
Rahman et al.^46^	SVM	Multiclass	500 images	93.3

An accuracy of 80%, 82.23%, and 84% were achieved using machine learning techniques such as SVM and kNN on the dataset used in this research^16,20,47^. For the fair performance comparison, we utilized ResNeXt, AlexNet, and VGG16 for the multiclass (superficial vs. deep dermal vs. full thickness) and binary class (graft vs. non-graft) classification. In addition, the same training setup and dataset were used to evaluate the model’s performance. In Table 7, we summarize the performance of multiclass classification, and in Table 7, the performance of graft and non-graft is discussed. In Table 6, we can see AlexNet achieved the lowest classification accuracy of 70.57%, Whereas ResNeXt obtained 84.31% and the proposed BuRnGANeXt50 achieved a classification accuracy of 98.14%.

**Table 7:** Evaluation of the presented model versus the previous model for multiclass classification.

Model	Recall (%)	F1-score (%)	Precision (%)	Accuracy (%)
ResNeXt	83.46	82.9	82.34	84.31
AlexNet	67.53	67.89	68.26	70.57
VGG16	73.27	75.02	76.87	76.32
BuRnGANeXt50	97.22	97.23	97.22	98.14

In Table 8, we can see that the precision and F1-score of the AlexNet are 73.14% and 71.62%, respectively. Slight improvement was noticed in VGG16. The second highest precision and F1-score achieved by ResNeXt. Whereas the proposed model achieved 99. 86% and 99.49% precision and recall values, respectively.

**Table 8:** Evaluation of the presented model versus the previous model for binary classification.

Model	Recall (%)	F1-score (%)	Precision (%)	Accuracy (%)
ResNeXt	85.15	85.30	85.46	86.35
AlexNet	70.18	71.62	73.14	75.64
VGG16	76.50	78.31	80.20	78.23
BuRnGANeXt50	99.14	99.49	99.86	99.48

The provided BuRnGANeXt50 model showed the best results for multiclass and binary class classification. In addition, the computation time per epoch and trainable parameters are very less, as shown in Table 9. The BuRnGANeXt50 model can be used for real-time applications and provide a second healthcare opinion.

**Table 9:** Comparison of the computation time per epoch.

Model	Time per epoch	Parameters
AlexNet	124 s	24 × 10^6^
VGG16	185 s	33 × 106
ResNeXt	175 s	23 × 106
BuRnGANeXt50	105 s	5 × 106

**Figure 9:**
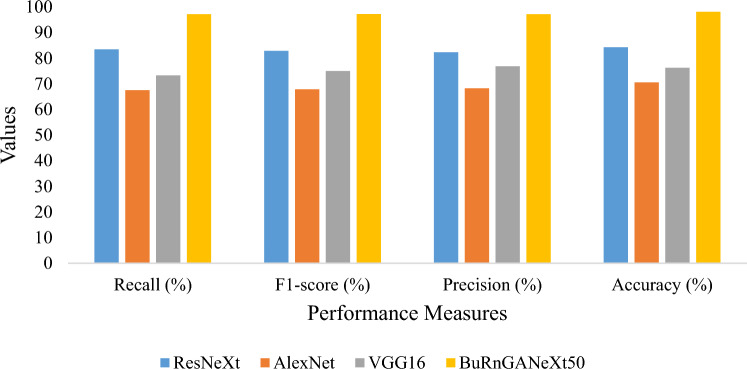
Bar plot-based comparison for multiclass classification.

**Figure 10:**
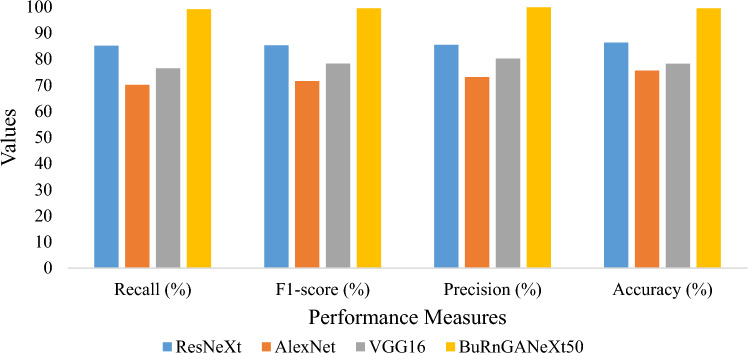
Bar plot-based comparison for binary classification.

We compare the performance of the proposed method and ResNeXt, AlexNet, and VGG16 for multiclass and binary class classification shown in Figs. 9 and 10, respectively. In Fig. 9, we can notice that all performance measure bars of the BuRnGANeXt50 are much higher than the other state-of-the-art methods. Similarly, in Fig. 10, we can notice ResNeXt performance measure bars are much better than the AlexNet and VGG16. However, the proposed method’s performance measures precision, recall, F1-score, and accuracy is much better than ResNeXt.

**Figure11:**
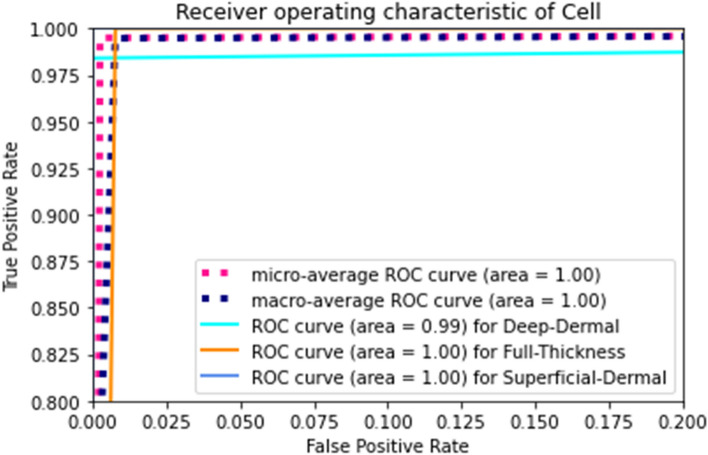
ROC curve of the multiclass burn diagnosis.

Furthermore, we plotted the ROC (Receiver Operating Characteristic) curve of the proposed method’s true positive rate and false positive rate for multiclass classification, shown in Fig. 11. We can notice that the ROC value of superficial and full thickness burn is 1, whereas the deep dermal burn is 0.99. This confirms the proposed BuRnGANeXt50 is highly sensitive for burn diagnosis.

### Limitations

The proposed model algorithm computation cost is still a challenge. In addition, the attention module provides only local dependencies of the features. That may reduce the performance in some scenarios.

## Conclusion

Burn diagnosis is timely and accurately is necessary to save the patient’s life. The traditional method of burn diagnosis is time-consuming, and the accuracy depends on the dermatologist’s expertise. Recent advancement in ML and DL in medical imaging has improved the accuracy and reduced the diagnosis time. However, ML-based methods require handcrafted features for model training that may reduce efficiency. Conversely, the Shallow DL method extracts features automatically but lacks the feature correlation dependency. We conducted experiments using AlexNet, VGG16, and ResNext. However, these models’ performance for classifying burns could be more optimal, and the computation costs are high due to high trainable parameters. The original ResNext performance is better compared to AlexNet and VGG16 due to the capability of capturing high-dimensional features. Many trainable parameters and activation functions make the model less reliable for real-time applications.

In this study, we proposed a modified residual network with less trainable parameters and an attention block for burn diagnosis. After extensive experiments, the convolution and filter size are optimized. Further, instead of ReLu activation, Leaky ReLu activation is utilized, which improves the convergence rate. The spatial attention module enables the model to focus on significant regions of interest, such as burn edges, blisters, and regions with varying degrees of injury. In the meantime, the channel attention module concentrates on crucial characteristics within each network layer, enabling the model to extract the most informative aspects from the input data. Combining spatial and channel attention mechanisms enables our model to learn discriminative patterns from burn images, resulting in superior diagnostic performance. The model’s performance for classifying burns based on degree and depth into three classes and binary class is much better than the state-of-the-art method. The precision and accuracy of the BuRnGANeXt50 for multiclass classification are 97.22% and 98.14%, respectively. Furthermore, the proposed model classifies the burn into graft and non-graft with a precision and accuracy of 99.86% and 99.48%, respectively. This confirms the model is highly sensitive for burn diagnosis and can provide a second opinion to a doctor. In addition, the model computation time per epoch is much less, making it suitable for real-time applications.

The computation time of the proposed is still a challenge that needs further improvement. In addition, the model needs to test on other diverse datasets and a real-time dataset for further evaluation. We found some images of deep dermal classified to full thickness due to similar texture and color characteristics. Furthermore, the results need to be evaluated by the healthcare expert. In future research, we will explore capturing the global relation of the features using a vision transformer-based model to improve the long-range dependency of the features. In addition, the extracted features can be optimized using nature-inspired algorithms to enhance the classification accuracy. Furthermore, a calibration technique can be applied to measure the model’s bias. Furthermore, addressing the challenges associated with model interpretability can be improved using a grad cam.

## Data Availability

The data supporting this study’s findings are available upon request from the corresponding authors.
